# Screening premorbid metabolic syndrome in community pharmacies: a cross-sectional descriptive study

**DOI:** 10.1186/1471-2458-14-487

**Published:** 2014-05-22

**Authors:** Maria Angeles Via-Sosa, Cristina Toro, Pere Travé, Marian A March

**Affiliations:** 1 Unit of Practice Pharmacy, Unidad de Prácticas Tuteladas, Faculty of Pharmacy, University of Barcelone, Catalonia, Spain; 2 Faculty of Pharmacy, University of Barcelona, Catalonia, Spain

**Keywords:** Community pharmacy, Screening, Metabolic syndrome, Cardiovascular risk, Cardiovascular disease, Diabetes mellitus

## Abstract

**Background:**

Premorbid metabolic syndrome (pre-MetS) is a cluster of cardiometabolic risk factors characterised by central obesity, elevated fasting glucose, atherogenic dyslipidaemia and hypertension without established cardiovascular disease or diabetes. Community pharmacies are in an excellent position to develop screening programmes because of their direct contact with the population.

The main aim of the study was to determine the prevalence of pre-MetS in people who visited community pharmacies for measurement of any of its five risk factors to detect the presence of other risk factors. The secondary aims were to study the presence of other cardiovascular risk factors and determine patients’ cardiovascular risk.

**Methods:**

Cross-sectional, descriptive, multicentre study. Patients meeting selection criteria aged between 18 and 65 years who visited participating community pharmacies to check any of five pre-MetS diagnostic factors were included.The study involved 23 community pharmacies in Catalonia (Spain). Detection criteria for pre-MetS were based on the WHO proposal following IDF and AHA/NHBI consensus. Cardiovascular risk (CVR) was calculated by Regicor and Score methods. Other variables studied were smoking habit, physical activity, body mass index (BMI), and pharmacological treatment of dyslipidemia and hypertension. The data were collected and analysed with the SPSS programme. Comparisons of variables were carried out using the Student’s T-test, Chi-Squared test or ANOVA test. Level of significance was 5% (0.05).

**Results:**

The overall prevalence of pre-MetS was 21.9% [95% CI 18.7-25.2]. It was more prevalent in men, 25.5% [95% CI 22.1-28.9], than in women, 18.6% [95% CI 15.5-21.7], and distribution increased with age. The most common risk factors were high blood pressure and abdominal obesity. About 70% of people with pre-MetS were sedentary and over 85% had a BMI ≥25 Kg/m^2^. Some 22.4% had two metabolic criteria and 27.2% of patients with pre-MetS had no previous diagnosis.

**Conclusions:**

The prevalence of pre-MetS in our study (21.9%) was similar to that found in other studies carried out in Primary Care in Spain. The results of this study confirm emergent cardiometabolic risk factors such as hypertension, obesity and physical inactivity.

Our study highlights the strategic role of the community pharmacy in the detection of pre-MetS in the apparently healthy population.

## Background

The great increase in the incidence of cardiovascular disease (CVD) and type 2 diabetes mellitus (T2DM) among patients throughout the developed and developing world means that physicians and other care providers have to be aware of the risk factors for these conditions. They should be able to identify patients at risk to initiate treatment to prevent these diseases [1]. Identifying individuals at risk of chronic diseases is the first step towards preventive measures [2].

Metabolic syndrome (MetS) is a cluster of cardiovascular risk factors. Although there are various definitions of metabolic syndrome, the syndrome’s common pathophysiology is insulin resistance and a prominent clinical feature of this syndrome is abdominal or central obesity [3]. The term “cardiometabolic risk” is now starting to gain greater acceptance as it includes the entire cluster of classic cardiovascular risk factors and other emerging factors such as those related to abdominal obesity and insulin resistence [4]. It is known that patients who develop CVD or T2DM have common histories of disorders of metabolic origin [1]. The criteria for identifying MetS includes five variables, namely, abdominal obesity, raised triglycerides, low high-density lipoproteins (HDL-cholesterol), elevated blood pressure and impaired fasting glucose state [5]. The lack of consensus on diagnostic criteria does not allow comparison of distinct MetS prevalence data. However, irrespective of the criteria used, the reality is that MetS is indeed highly prevalent, especially in developed countries [5,6]. Initial studies in the U.S recognise MetS as a principal health problem in developed countries with considerable social and economic consequences [6].

Some years ago, International Diabetes Federation (IDF) and American Heart Association/National Heart, Lung and Blood Institute (AHA/NHLBI) representatives attempted to resolve the remaining differences between definitions of metabolic syndrome [4]. They agreed that abdominal obesity should not be a prerequisite for diagnosis but that it is 1 of 5 criteria, and that the presence of any 3 of 5 risk factors constitutes a diagnosis of metabolic syndrome.

Concerning the metabolic syndrome concept, in 2010 the World Health Organization (WHO) proposed that MetS should be considered a premorbid condition [7]. Therefore, screenings for pre-MetS should exclude individuals with established diabetes or known cardiovascular disease [7]. Exclusion of these patients is the main difference between the metabolic syndrome and premorbid metabolic syndrome concepts.

Nowadays, pre-MetS has been receiving attention not only in hospital-based medicine but also in primary care (PC) and the community pharmacy. Since epidemiological studies [2,6,8,9] suggest that >25% of the general population will gradually develop insulin resistance, it would appear that this pathology will increasingly be diagnosed and treated within the ambit of PC. Moreover, screening will be included in the list of priorities of various public health-care authorities [6].

Community pharmacies are in an excellent position to develop screening programmes to identify individuals at risk of developing CVD or T2DM because of their contact with citizens whether they are ill or not [10]. The quality of care offered by the pharmacist, who can also provide health interventions to individuals [10], allows the extension of individual clinical interventions to a larger percentage of the population that does not visit the doctor or seek primary or hospital care.

The main aim of the study was to determine the prevalence of pre-MetS in people who visited community pharmacies for measurement of any of its five risk factors to detect the presence of other risk factors. The secondary aims were to study the presence of other cardiovascular risk factors and determine patients’ cardiovascular risk.

## Methods

### Design

Cross-sectional, descriptive, multicentre study to determine the prevalence of premorbid metabolic syndrome in patients who visit community pharmacies.

### Study population

**Figure 1:**
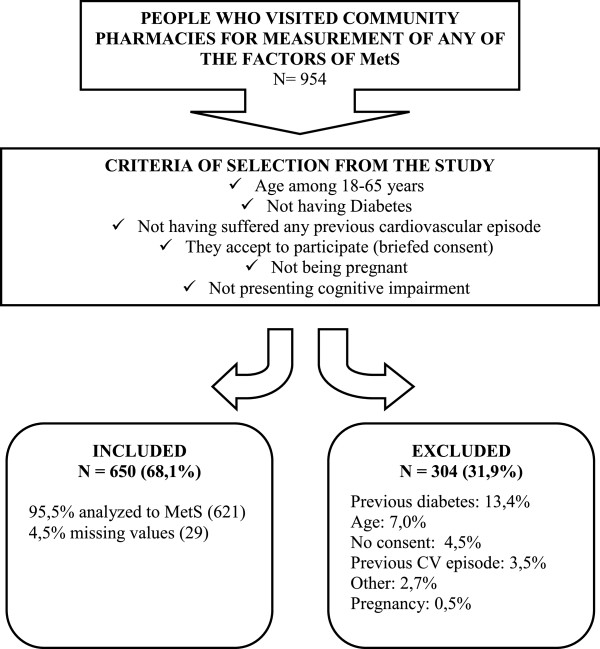
**General study schema.** Cross-sectional, descriptive, multicentre study to determine the prevalence of premorbid metabolic syndrome in patients who visit community pharmacies.

Individuals aged between 18 and 65 years who visited the participating community pharmacies to check any of the five pre-MetS diagnostic factors during the 11-month study period (Nov’, 2009-Oct’, 2010) were recruited sequentially. Those patients who spontaneously visited the community pharmacy to measure any of the five risk factors were invited to participate in the study. The sample consisted of 650 patients meeting the following inclusion criteria: not having diabetes, not having suffered any previous cardiovascular episode, not being pregnant, not presenting cognitive impairment that interferes with understanding of the study, and agreeing to participate in it (Figure 1). Written informed consent was obtained for all participants (no children were studied). The Germans Trias i Pujol Hospital Ethics Committee revised and approved the study (EO-12-038). This sample size allows us to estimate the prevalence of pre-MetS with a precision level of ±3% and an α risk of 0.05 for an estimated theoretical pre-MetS prevalence of 20%.

### Setting

The study involved 23 volunteer community pharmacies situated to the northeast (Barcelona Nord and Maresme) and the southwest (Costa Ponent) of Barcelona City, Catalonia (Spain), from a total of 553 community pharmacies who provide pharmaceutical services to approximately 2,250,000 people. Participating pharmacies had accreditation to supervise senior pharmacy students from the University of Barcelona. The pharmacists and the senior students received 4-hour training session prior to the study.

### Variables studied

All data were collected by pharmacists using a data collection sheet. Age, gender, smoking habit, physical activity and pharmacological treatment of pre-MetS diagnostic factors were self-reported and noted during a clinical interview with the patients studied at the pharmacies. Physical activity was evaluated according the Baleares Primary Care Guide [11]. According to this guide, the patient was not considered sedentary if he or she engaged in physical exercise for 30 minutes or more, four or five times per week. Physical exercise was defined as any activity which caused sweating [11]. Smoking habit was considered absent when the participant had not smoked during the previous year. Anthropometrics were obtained with the participant wearing light clothing and barefoot. Body mass index (BMI) (Kg/m^2^) was calculated through weight (Kg) and height (m) measured by calibrated digital scales and stadiometers [12]. Fasting glucose (FG) (mg/dl), total cholesterol (mg/dl) and triglycerides (mg/dl) were measured with Reflotron® (Roche Diagnostics) after an overnight fast (lasting at least 8 hours) and HDL-cholesterol (mg/dl) was obtained from a Blood Test Report taken within the previous 3 months. Abdominal obesity (AO) was determined by the measurement of waist circumference (WC) midway between the inferior margin of the ribs and the superior border of the iliac crest. Systolic and diastolic blood pressure (SBP and DBP) were measured twice with validated and calibrated electronic sphygmomanometers after 5 minutes rest. Cardiovascular risk was calculated according to Framingham-Regicor [13,14] predictive equations calibrated for Girona (Spain) and according to the Score [13] project for European population at low risk. Framingham-Regicor risk tables have been adapted and validated with data from the population of Girona (Spain) in the VERIFICA study [14,15].

To determine the prevalence of pre-MetS in our study, we used the MetS defining criteria according to the most recent IDF and AHA/NHLBI consensus [4] and the WHO [7] premorbid condition, i.e., individuals not diagnosed with cardiovascular disease or diabetes and having at least three of the five risk factors: Waist circumference (WC): ♂ ≥ 102 cm or ♀ ≥ 88 cm (for Europeans); triglycerides (TG): ≥150 mg/dl (1.7 mmol/L) or taking lipid-regulating medication; high density lipoproteins (HDL-cholesterol): ♂ <40 mg/dL (1.0 mmol/L), ♀ <50 mg/dL (1.3 mmol/L) or drug treatment for reduced HDL-cholesterol; blood pressure (BP): ≥130/85 mm Hg or taking antihypertensive medication, and fasting glucose (FG): ≥100 mg/dL (5.6 mmol/L).

### Statistical analysis

Statistical analysis was conducted with the SPSS programme (version 15.0). The variables measured on a quantitative scale are expressed as meanss (±Standard Deviation) and those variables measured on a qualitative scale are expressed as percentages (95% Confidence Interval). Comparison between quantitative variables was carried out using the Student’s T-Test when they were dichotomous, and with the ANOVA test where there were more than two categories. Comparison between qualitative variables was performed with the Chi-Squared test. The significance level was set at 5%.

## Results

### Description of study population

Figure 1 shows the general outline of the study, the number of patients who were excluded and the distinct reasons for exclusion.

Table 1 (n = 650) summarises the baseline characteristics of the included patients segregated by gender. A total of 51.8% (337) of the participants were female and the overall mean age of included patients was 48.4 ± 12.5 years. There were no statistically significant differences between the men’s and women’s mean ages.

**Table 1:** Baseline characteristics of studied population according to gender

	***Total n = 650***	***Men n = 313***	***Women n = 337***	***p***
Age^1^ (years)	48.4 (SD = 12.5)	48.9 (SD = 12.5)	48.0 (SD = 12.5)	0.341
Waist C^1^ (cm)	92.3 (SD = 13.6)	97.7 (SD = 11.6)	87.1 (SD = 13.3)	<0.001
BMI^1^ (kg/m^2^)	26.4(SD = 4.5)	27.1 (SD = 4.1)	25.7 (SD = 4.7)	<0.001
Fasting glucose^1^ (mg/dl)	93.6 (SD = 14.6)	95.5 (SD = 14.6)	91.9 (SD = 14.3)	0.002
Total cholesterol^1^ (mg/dl)	204.9 (SD = 39.6)	201.8 (SD = 41.2)	207.8 (SD = 37.8)	0.051
HDL-cholesterol^1^ (mg/dl)	58.7 (SD = 14.7)	55.4 (SD = 13.4)	61.8 (SD = 15.3)	<0.001
Triglyceride^1^ (mg/dl)	123.0 (SD = 83.4)	138.7 (SD = 102.6)	108.3 (SD = 56.5)	<0.001
SBP^1^ (mm Hg)	127.2 (SD = 16.2)	130.5 (SD = 16.1)	124.2 (SD = 15.7)	<0.001
DBP^1^ (mm Hg)	77.3 (SD = 10.5)	79.5 (SD = 10.2)	75.2 (SD = 10.3)	<0.001
CVR^1^ (Score)	1.5 (SD = 1.9)	2.4 (SD = 2.1)	0.8 (SD = 1.1)	<0.001
CVR^1^ (Regicor)	3.4 (SD = 2.3)	3.9 (SD = 2.6)	2.9 (SD = 1.9)	<0.001
AC drugs^2^	30.0 (26.5-33.5)	33.5 (28.3-38.7)	26.7 (22.0-31.4)	0.060
AH drugs^2^	32.3 (28.7-35.9)	40.3 (34.9-45.7)	24.9 (20.3-29.5)	<0.001
Smoking habit^2^	30.0 (26.5-33.5)	34.8 (29.5-40.1)	25.5 (20.8-30.2)	0.010
Physical inactivity^2^	58.5 (54.7-62.3)	52.7 (47.2-58.2)	63.8 (58.7-68.9)	0.005

p ≤ 0.05 is considered to be statistically significant.

^1^Data are expressed as means (Standard Deviation).

^2^Data are expressed as percentages (95% confidence interval).

*Waist C* waist circumference; *BMI* body mass index; *SBP* systolic blood pressure; *DBP* diastolic blood pressure; *CVR* cardiovascular risk; *AC Drugs* pharmacological treatment of hypercholesterolemia or hypertriglyceridemia; *AH Drugs* pharmacological treatment of hypertension.

The mean body mass index (BMI) was 26.4 Kg/m^2^ [95% CI 26.0-26.7] and there were statistically significant differences by gender (27.1 Kg/m^2^ [95% CI 26.7-27.6] in men and 25.7 Kg/m^2^ [95% CI 25.2-26.2] in women). Some 42.3% of the study population had normal weight (BMI < 25), 39.5% were overweight (25 ≤ BMI < 30) and 18.2% were obese (BMI ≥ 30). 58.5% of the participants were considered physically inactive and this was statistically more prevalent in women than in men.

Some 49.8% (323) were receiving pharmacological treatment for at least one of the five pre-MetS diagnostic factors. About 30% of participants received pharmacological treatment for hypertension or dyslipidaemia and 13.4% (87) for both.

**Figure 2:**
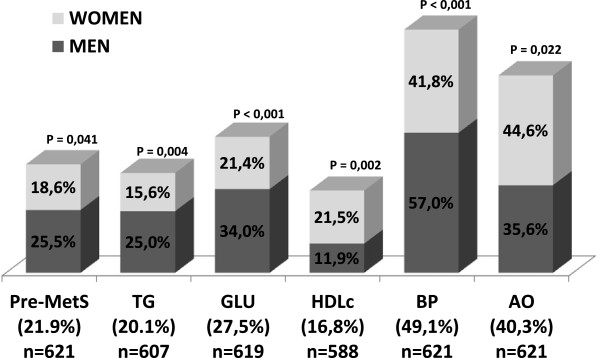
**Prevalence of premorbid metabolic syndrome based on the WHO proposal and their five diagnostic factors by gender.** Data are expressed as percentages. Pre-MetS: premorbid metabolic syndrome; TG: Triglycerides; GLU: fasting glucose; HDLc: HDL-cholesterol; BP: blood pressure; AO: abdominal obesity.

Of these 650 patients, 29 (4.5%) were excluded from the sample due to a lack of more than two diagnostic risk-factor values making it difficult to estimate a diagnosis for pre-MetS in these individuals. Triglycerides and HDL-cholesterol were the most difficult values to collate.The pre-MetS risk-factor prevalences were: elevated blood pressure in 49.1% (305), abdominal obesity in 40.3% (250), elevated fasting glucose in 27.5% (170), elevated triglycerides in 20.1% (122) and low HDL-cholesterol in 16.8% (99). All risk factors were more prevalent in men than women except abdominal obesity and HDL-cholesterol while the differing percentages between the genders were statistically significant (Figure 2).

Only 20.5% (127) of study population did not present any of the five pre-MetS diagnostic criteria risk factors, 31.7% (197) presented one of these metabolic criteria and 22.4% (139) had two. Regarding the 139 participants with two risk factors, 66.2% (92) had altered blood pressure and 59.0% (82) abdominal obesity.

Among the study participants, we found a high correlation between abdominal obesity and BMI.

### The prevalence of premorbid metabolic syndrome in the study population

The prevalence of pre-MetS was 21.9% [95% CI 18.7-25.2] and was more prevalent in the men 25.5% [95% CI 22.1-28.9] than in the women 18.6% [95% CI 15.5-21.7%] studied (Figure 2). Some 59.6% of patients with pre-MetS had three diagnostic criteria risk factors, 35.3% had four and only 5.1% had all five risk factors.

Of the 136 patients who had pre-MetS, 37 (27.2%) had not been previously diagnosed with dyslipidaemia or hypertension.

Table 2 (n = 621) shows information on the quantitative variables studied in two groups: those with and without pre-MetS. We should point out that all of these variables were statistically different in the two groups. Participants with pre-MetS were older than patients without it. Furthermore, people with this syndrome also presented higher values in other risk factors not included in the diagnostic criteria (BMI, total cholesterol, CVR Score and CVR Regicor) than participants without pre-MetS.

**Table 2:** Quantitative variables of the subjects as a function of the diagnosis of premorbid metabolic syndrome based on the WHO proposal

	***Total n = 621***	***Pre-MetS n = 136***	***Non Pre-MetS n = 485***	***p***
Age (years)	48.5 (SD = 12.4)	54.5 (SD = 9.2)	46.8 (SD = 12.7)	<0.001
Waist C (cm)	92.0 (SD = 13.6)	103.3 (SD = 11.8)	88.8 (SD = 12.3)	<0.001
BMI (kg/m^2^)	26.3 (SD = 4.5)	29.8 (SD = 4.6)	25.3 (SD = 3.9)	<0.001
Fasting glucose(mg/dl)	93.7 (SD = 14.3)	105.5 (SD = 17.9)	90.3 (SD = 10.9)	<0.001
Total cholesterol (mg/dl)	205.3 (SD = 39.9)	219.5 (SD = 41.1)	201.4 (SD = 38.7)	<0.001
HDL-cholesterol (mg/dl)	58.8 (SD = 14.7)	49.2 (SD = 13.4)	61.5 (SD = 14.0)	<0.001
Triglyceride (mg/dl)	122.7 (SD = 83.4)	189.3 (SD = 132.7)	103.8 (SD = 48.8)	<0.001
SBP (mm Hg)	126.8 (SD = 16.2)	138.3 (SD = 15.5)	123.6 (SD = 14.9)	<0.001
DBP (mm Hg)	77.0 (SD = 10.4)	82.7 (SD = 10.7)	75.4 (SD = 9.6)	<0.001
CVR (Score)	1.5 (SD = 1.9)	2.5 (SD = 2.3)	1.2 (SD = 1.5)	<0.001
CVR (Regicor)	3.3 (SD = 2.3)	4.8 (SD = 2.6)	2.8 (SD = 1.9)	<0.001

p ≤ 0.05 is considered to be statistically significant.

Data are expressed as means (Standard Deviation).

*Pre-MetS* premorbid metabolic syndrome; *BMI* body mass index; *SBP* systolic blood pressure; *DBP* diastolic blood pressure; *CVR* cardiovascular risk.

Table 3 (n = 621) shows the percentages of the qualitative variables studied divided into groups corresponding to those with and without pre-MetS. All variables showed statistically significant differences between groups except the number of smokers.

**Table 3:** Qualitative variables of the subjects as a function of the diagnosis of premorbid metabolic syndrome based on the WHO proposal

		***Total n = 621***	***Pre-MetS n = 136***	***Non Pre-MetS n = 485***	***p***
Sex	♂	48.0 (44.1-51.9)	55.9 (47.6-64.2)	45.8 (41.4-50.2)	
	♀	52.0 (48.1-55.9)	44.1 (35.8-52.4)	54.2 (49.8-58.6)	<0.001
AC drugs		30.0 (26.4-33.6)	49.3 (40.9-57.7)	24.5 (20.7-28.3)	<0.001
AH drugs		32.7 (29.0-36.4)	49.3 (40.9-57.7)	28.0 (24.0-32.0)	<0.001
Smoking habit		30.1 (26.5-33.7)	27.9 (20.4-35.4)	30.7 (26.6-34.8)	0.597
Physical inactivity		58.9 (55.0-62.8)	69.9 (62.2-77.6)	55.9 (51.5-60.3)	0.004
BMI	<25	42.4 (38.5-46.3)	12.5 (7.0-18.1)	50.7 (46.6-55.2)	
	≥25	57.6 (53.7-61.5)	87.5 (81.9-93.1)	49.3 (44.9-53.7)	<0.001

p ≤ 0.05 is considered to be statistically significant.

Data are expressed as percentages (95% Confidence Interval).

*Pre-MetS* premorbid metabolic syndrome; *AC* drugs: pharmacological treatment of hypercholesterolemia or hypertriglyceridemia; *AH Drugs*: pharmacological treatment of hypertension.

Table 4 (n = 621) shows that the prevalence of pre-MetS increased with age. This prevalence was about four times higher in older people than in younger patients. Over two-thirds of participants with pre-MetS were more than 53 years old. In addition, it was observed that the prevalence by the different age groups classified by gender showed no statistically significant differences.

**Table 4:** Sex and age-specific prevalence of premorbid Metabolic Syndrome based on WHO proposal

	***Prevalences of Pre-MetS***			
	***Total (%)***	***Men (%)***	***Women (%)***	***p***
Age ranges:				
18-39 years	8.7 (14/161)	10.7 (8/75)	7.0 (6/86)	0.419
40-52 years	17.9 (30/168)	25.0 (21/84)	10.7 (9/84)	0.026
53-60 years	28.1 (43/153)	34.9 (22/63)	23.3 (21/90)	0.144
61-65 years	35.3 (49/139)	32.9 (25/76)	38.1 (24/63)	0.594
Total	21.9 (136/621)	25.5 (76/298)	18.6 (60/323)	0.041

**Figure 3:**
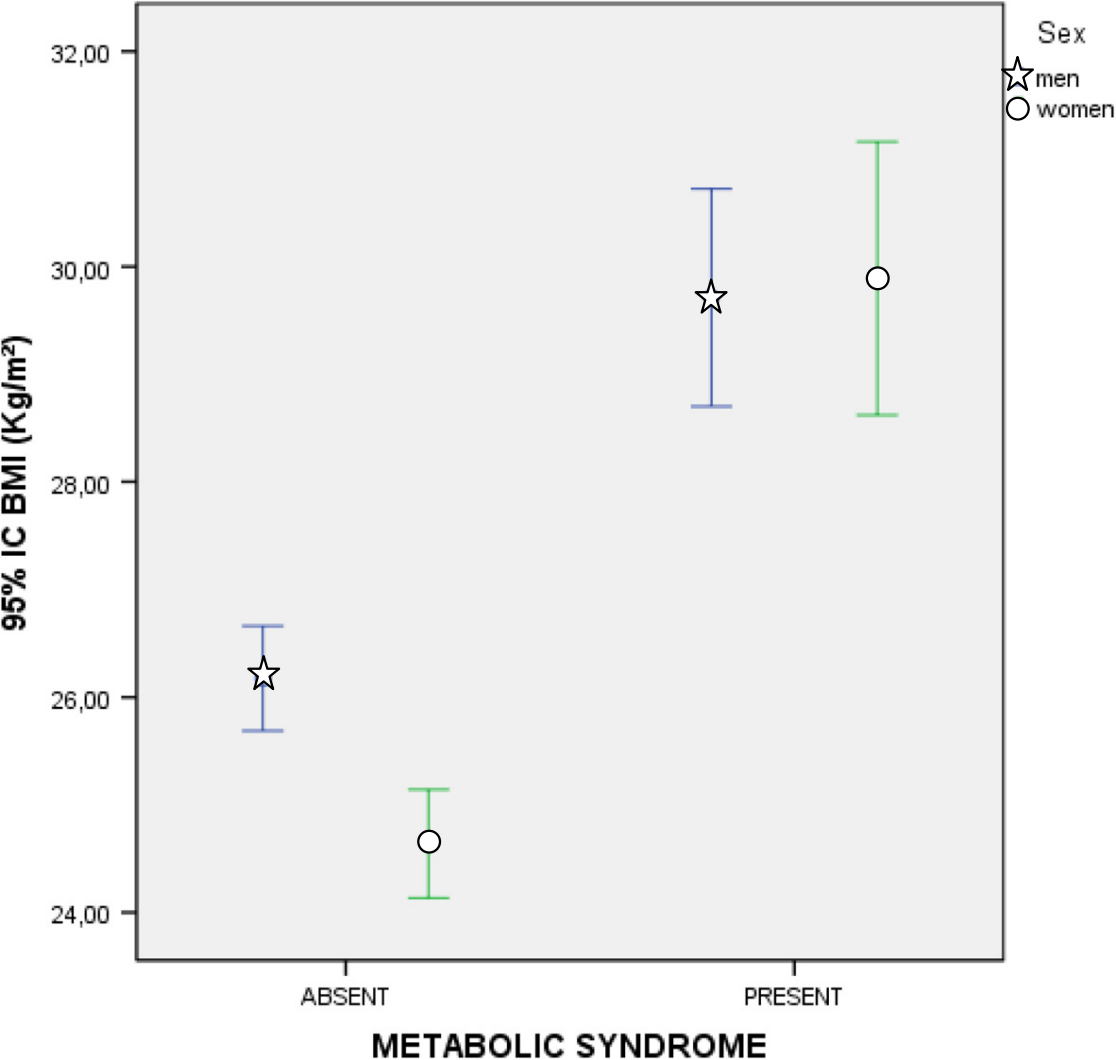
**Error Bars (95% CI) of body mass index according to the diagnosis of premorbid metabolic syndrome and gender.** CI: Confidence Interval; BMI: body mass index.

**Figure 4:**
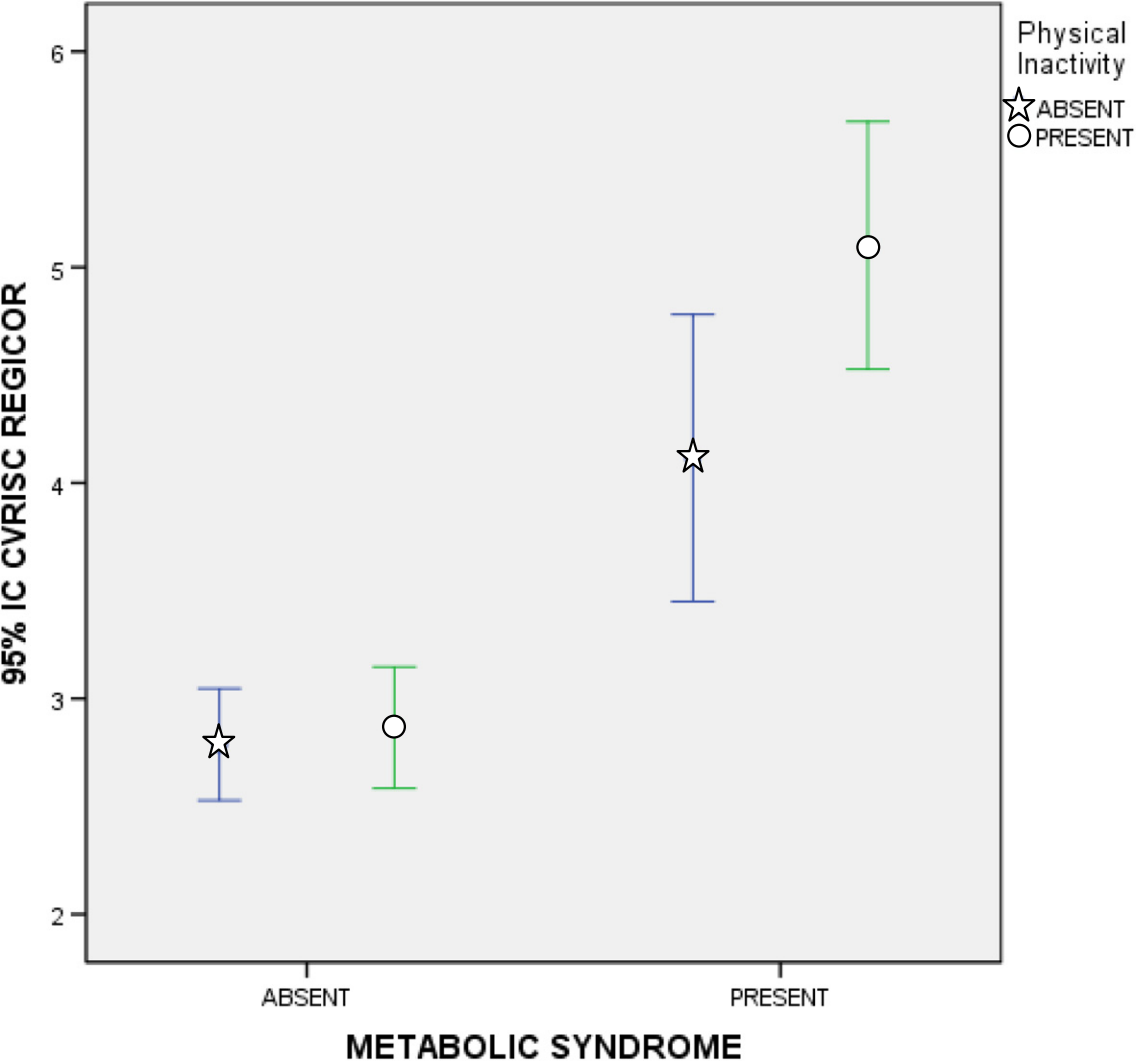
**Error Bars (95% CI) of Cardiovascular Risk (Regicor) according to the diagnosis of premorbid metabolic syndrome and physical inactivity.** CI: Confidence Interval.

**Figure 5:**
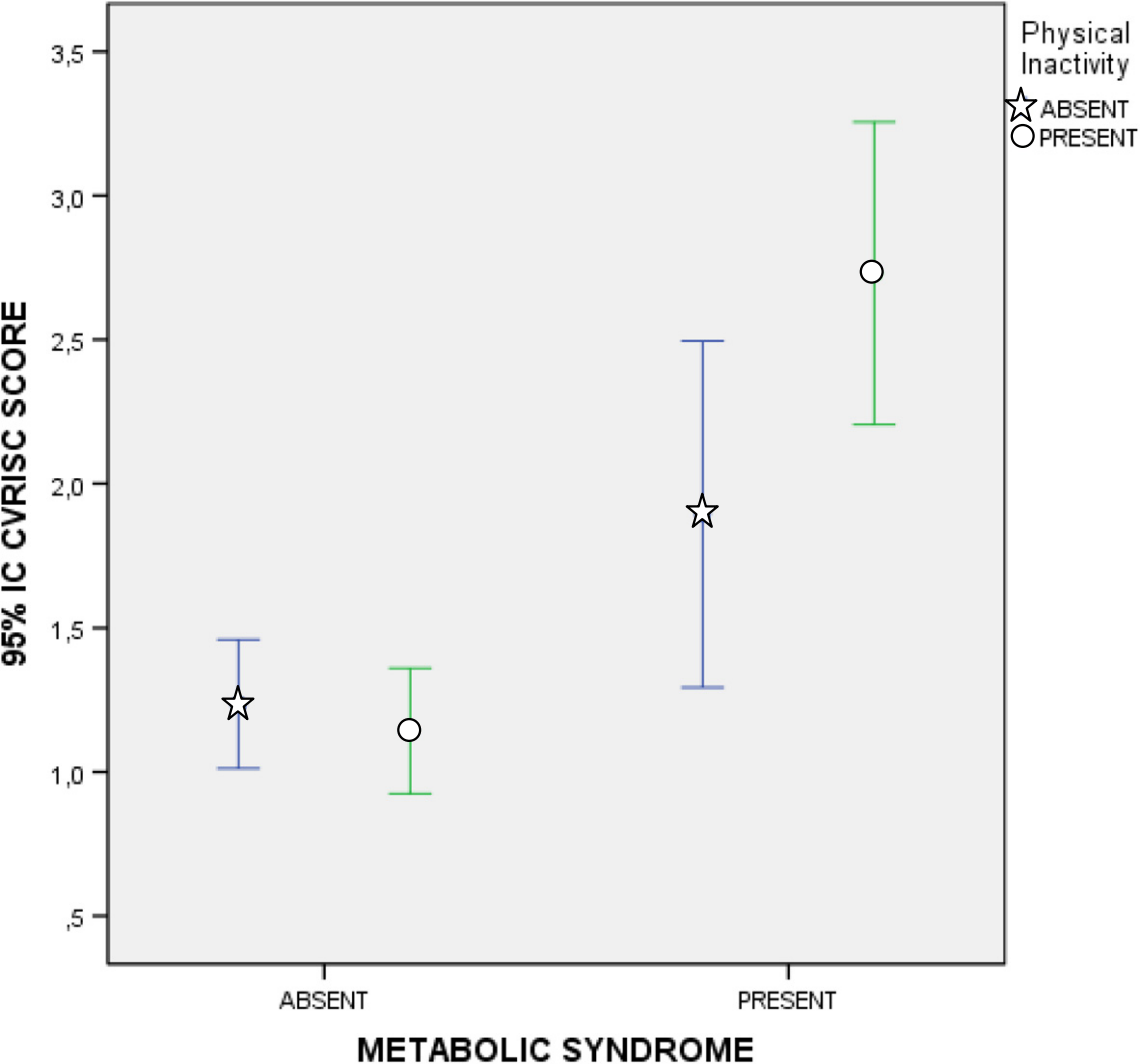
**Error Bars (95% CI) of Cardiovascular Risk (Score) according to the diagnosis of premorbid metabolic syndrome and physical inactivity.** CI: Confidence Interval.

As mentioned above, Body Mass Index (BMI) is a cardiometabolic risk factor not included in the pre-MetS diagnostic criteria. In Table 3, it can be seen that 87.5% of participants with pre-MetS were overweight or obese, that is to say, they had a BMI ≥ 25. Table 5 shows the statistically significant differences observed in all quantitative variables between subjects with BMI < 25 and those with BMI ≥ 25. When comparing individuals with a BMI between BMI ≥ 25 and BMI ≥ 30, almost none of the parameters studied showed statistically significant differences. However, statistically significant differences were observed in some MetS diagnostic criteria risk factors (triglycerides, waist circumference and systolic blood pressure).Figure 3 shows the BMI error bars by gender according to the presence or absence of pre-MetS. It was determined that men and women who presented pre-MetS had similar BMI. However, men without pre-MetS had a significantly higher BMI than women without pre-MetS.Figures 4 and 5 show the cardiovascular risk error bars (Regicor and Score, respectively) according to the presence or absence of pre-MetS and physical inactivity. In both figures, we can see that sedentary patients with pre-MetS had a higher cardiovascular risk than physically active patients. This difference was less significant in patients without pre-MetS.

**Table 5:** Studied variables of the subjects according to BMI categories

	**Body mass index (Kg/m**^**2**^**)**				
	***BMI < 25 n = 263***		***25 ≤ BMI < 30 n = 245***		***BMI ≥ 30 n = 113***
Age (Years)	44.0 (SD = 13.6)		51.4 (SD = 10.4)		52.4 (SD = 10.2)
p		<0.001		0.765	
Fasting glucose (mg/dl)	89.2 (SD = 10.9)		96.0 (SD = 15.8)		99.0 (SD = 14.8)
p		<0.001		0.132	
Total cholesterol (mg/dl)	198.2 (SD = 40.5)		210.4 (SD = 39.5)		211.1 (SD = 37.1)
p		0.001		0.986	
Triglycerides (mg/dl)	99.5 (SD = 55.1)		131.9 (SD = 81.0)		155.0 (SD = 120.8)
p		<0.001		0.032	
HDL-cholesterol (mg/dl)	62.7 (SD = 15.3)		56.1 (SD = 13.8)		55.4 (SD = 13.3)
p		<0.001		0.913	
Waist circumference (cm)	82.1 (SD = 9.7)		95.3 (SD = 8.9)		107.3 (SD = 11.0)
p		<0.001		<0.001	
SBP (mm Hg)	121.9 (SD = 15.3)		128.6 (SD = 15.7)		134.4 (SD = 15.8)
p		<0.001		0.003	
DBP (mm Hg)	74.3 (SD = 10.2)		78.6 (SD = 9.5)		80.1 (SD = 10.7)
p		<0.001		0.396	
CVRSCORE (%)	1.0 (SD = 1.3)		1.8 (SD = 2.1)		1.8 (SD = 2.0)
p		<0.001		0.991	
CVR REGICOR (%)	2.6 (SD = 1.7)		3.7 (SD = 2.5)		3.8 (SD = 2.4)
p		<0.001		0.964	

p ≤ 0.05 is considered to be statistically significant.

Data are expressed as means (Standard Deviation).

*BMI* body mass index; *SBP* systolic blood pressure; *DBP* diastolic blood pressure; *CVR* cardiovascular.

## Discussion

The main advantages of these screenings for primary prevention carried out in community pharmacies are the proximity to the patients, the availability of quick, easy-to-apply screening tests and, where necessary, the option of carrying out a pharmaceutical intervention on the spot. A further positive aspect is that it is not only ill patients who visit the community pharmacy to pick up medication but also apparently healthy individuals who have other reasons to attend such as weight or arterial pressure control.

In our study, slightly more than half (50.2%) of study participants had not received any pharmacological treatment for any of the five pre-MetS diagnostic factors, that is, they were apparently healthy participants. Among these patients who were not taking any medication for any of the factors, 12.1% had pre-MetS. Moreover, a little more than a quarter (27.2%) who had pre-MetS had not been previously diagnosed with dyslipidemia or hypertension. These two findings highlight the value of the screening service provided by community pharmacies.

To date, the recent concept of metabolic syndrome as a premorbid condition [7] has rarely been used in prevalence studies [2,5,6,8,9]. According to the literature worldwide, there are few published studies using this new concept [16,17]. One of them is the DARIOS [16] study which included approximately 24,000 Spanish patients in primary care and found a higher prevalence of pre-MetS (24%) than in our study (21.9% [95% CI 18.7-25.2]). A possible explanation for this could be the older age range (35–74 years). As in our study, pre-MetS was more prevalent in men (26% [95% CI 23–28]) than in women (24% [95% CI 21–27]). The distribution of pre-MetS by gender and age was similar in both studies. In our study, the prevalence of pre-MetS was higher in men up to 60 years old and above 61 years it was higher in women. In the DARIOS [16] study, prevalence was higher in men up to 54 years of age, similar in men and in women between 55 and 64 years, and above 65 years it was higher in women. As the DARIOS [16] study also mentioned, an important factor that could explain the higher prevalence in older women is the menopause. The reduction in female hormonal production involves alterations in the lipid profile and increases abdominal obesity. These cardiometabolic risk factors may all be accompanied by an increase in insulin resistance [18,19].

Another study which considered the premorbid condition is the HERMEX [19] study. It included about 2,800 Spanish patients in primary care in Badajoz (Spain). The participants in this study were randomly recruited in accordance with the population census and were between 25 and 79 years old. The estimated prevalence of pre-MetS in the HERMEX study was 20.8% (95% CI: 19.3-22.3%). In common with our findings, it was more prevalent in men (23.5% [95% CI 21.2-25.8]) than in women (18.5% [95% CI 16.5-20.4]) and distribution by gender and age was very similar to that found in the DARIOS [16] study.

In contrast, there are many studies of MetS prevalence (with diabetic patients and previous CVD included) which obtained distinct prevalence values depending on diagnostic criteria (IDF, NCEP-ATPIII, etc.) and almost all were higher than in our study [6,8,9,20,21]. The exclusion of patients with T2DM or previous CVD significantly decreases the prevalence of MetS and, at the same time, allows enhancement of primary prevention of cardiometabolic diseases [7,22,23].

We were able to determine the most common pre-MetS risk factors in people who visit community pharmacies. As in the DARIOS [16] and HERMEX [17] studies, the most prevalent risk factors were hypertension and abdominal obesity, followed by raised fasting glucose, increased triglycerides and, finally, low HDL-cholesterol. As in these two studies, elevated fasting glucose and raised triglycerides were more common in men and low HDL-cholesterol and abdominal obesity were more common in women. In contrast to the DARIOS [16] and HERMEX [19] studies, we observed statistically significant differences in raised blood pressure between men and women. It was more prevalent in men than in women.

Unfortunately, in comparative studies [16,17], other risk factors such as physical inactivity or BMI with premorbid condition were not studied, that is, patients with T2DM or previous CVD were not excluded to determine the prevalences of these other risk factors.

There are many risk factors highly associated with poor nutrition and sedentary lifestyle [24,25]. In community pharmacies, the pharmacist may suggest several non-pharmacological interventions such as nutritional and lifestyle modifications [26,27]. The most suitable subjects for these interventions are those who have pre-MetS (21.9%) and patients with two of the five diagnostic criteria (22.4%).

On the one hand, the pharmacist can promote primary prevention of CVD and T2DM through encouraging a healthy lifestyle. The pharmacist can provide programmes to promote regular physical activities [28,29], mainly aerobic activities [30], to decrease body adipose tissue deposits, reduce blood pressure and increase insulin sensivity [31,32]. The pharmacist can also offer nutritional counselling for weight loss [33-35].

On the other hand, the pharmacist should promote secondary prevention in patients with diabetes, hypertension or dyslipidemia [35,36]. In these patients, health education at the community pharmacy has a very important role. As mentioned previously, some possible interventions that can be carried out on these patients include: design of an exercise plan, recommendations for individualised dietary measures, helping patients to give up smoking, identification of possible errors in medication, and advocacy of treatment adherence [35-38]. Diabetic patients can be offered routine checks of distinct biochemical parameters such as blood glucose or glycated haemoglobin [36]. They can also be informed about the acute or chronic complications of diabetes and guidelines for the correct handling and administration of anti-diabetic medication such as insulin [36]. In patients with arterial hypertension, it is very important to monitor arterial pressure and restrict consumption of salt in food [38,39]. The community pharmacy can also recommend some dietary health measures to those patients who present with dyslipidemia, such as a reduction in consumption of fats or a physical exercise programme [40]. Many studies demonstrate that all these interventions recommended by pharmacists allow better control of these illnesses and significantly reduce the risk of suffering a cardiovascular event [35,37-39].

The main aim for patients with pre-MetS is to maintain a normal weight. The pharmacist should recommend that patients with pre-MetS have a healthy, balanced diet. It is vital that these patients increase their intake of fresh fruit and vegetables and avoid food and drinks which are rich in sugar and fats, particularly saturated fats [34,35]. In our study, almost 90% of patients with pre-MetS were overweight or obese and had higher values in all studied variables than patients with a normal weight (BMI < 25). We found an optimal cutoff point in BMI of 25 Kg/m^2^ that shows the studied variables in the normal range. According to these results, BMI could be included as a pre-MetS diagnostic criteria risk factor. However, BMI is highly correlated with waist circumference, one of five included risk factors and, in addition, evidence has shown that waist circumference is a better body weight variable for predicting cardiometabolic diseases [41,42].

### Limitations

The present study does not attempt to extrapolate the results to the target population but rather to estimate the prevalence of pre-MetS in patients who regularly attend community pharmacies. Methodologically, this study has some biases:

•The non-inclusion of patients over 65 with greater prevalence of this syndrome.

•Recruitment of individuals from a pharmacy probably includes a similar population to that included in epidemiological studies from primary care. It is logical to assume that more ill patients will go to buy medicines than healthy people, although sometimes the healthy relatives buy the drugs.

•Low patient recruitment considering the involvement of 23 centres over a year which represents an average rate of 1.6 patients per pharmacy/per week.

## Conclusions

The prevalence of pre-MetS in our study (21.9%) was very similar to that found in other studies carried out in Primary Care in Spain. The results of this study confirm emergent cardiometabolic risk factors such as hypertension, obesity and physical inactivity.

Our study highlights the strategic role of the pharmacist and the community pharmacy in the detection of cardiometabolic risk factors in the apparently healthy population.

## Abbreviations

Pre-MetS: Premorbid metabolic syndrome; CVD: Cardiovascular diseases; T2DM: Type 2 diabetes mellitus; BMI: Body-mass index; HDLc: HDL-cholesterol; FG: Fasting glucose; WC: Waist circumference; TG: Triglycerides; AO: Abdominal obesity; SBP: Systolic blood pressure; DBP: Diastolic blood pressure; CVR: Cardiovascular risk.

## Competing interests

The authors declare that they have no competing interests.

## Authors’ contributions

MAVS wrote the article, contributed to discussion, researched data and edited the article. CT wrote the article, contributed to the discussion and edited the article. PT contributed to the discussion, researched data and reviewed the paper. MAM wrote the article, contributed to the discussion and reviewed the paper. All authors approved the final draft of the manuscript.

## Pre-publication history

The pre-publication history for this paper can be accessed here:

http://www.biomedcentral.com/1471-2458/14/487/prepub
